# *Ehrlichia chaffeensis* and Its Invasin EtpE Block Reactive Oxygen Species Generation by Macrophages in a DNase X-Dependent Manner

**DOI:** 10.1128/mBio.01551-17

**Published:** 2017-11-21

**Authors:** Omid Teymournejad, Mingqun Lin, Yasuko Rikihisa

**Affiliations:** Department of Veterinary Biosciences, The Ohio State University, Columbus, Ohio, USA; University of Chicago

**Keywords:** DNase X, *Ehrlichia*, EtpE, macrophages, neutrophils, reactive oxygen species

## Abstract

The obligatory intracellular pathogen *Ehrlichia chaffeensis* lacks most genes that confer resistance to oxidative stress but can block reactive oxygen species (ROS) generation by host monocytes-macrophages. Bacterial and host molecules responsible for this inhibition have not been identified. To infect host cells, *Ehrlichia* uses the C terminus of its surface invasin, entry-triggering protein of *Ehrlichia* (EtpE; EtpE-C), which directly binds the mammalian cell surface receptor glycosylphosphatidylinositol-anchored protein DNase X. We investigated whether EtpE-C binding to DNase X blocks ROS production by mouse bone marrow-derived macrophages (BMDMs). On the basis of a luminol-dependent chemiluminescence assay, *E. chaffeensis* inhibited phorbol myristate acetate (PMA)-induced ROS generation by BMDMs from wild-type, but not DNase X^−/−^, mice. EtpE-C is critical for inhibition, as recombinant EtpE-C (rEtpE-C)-coated latex beads, but not recombinant N-terminal EtpE-coated or uncoated beads, inhibited PMA-induced ROS generation by BMDMs from wild-type mice. DNase X is required for this inhibition, as none of these beads inhibited PMA-induced ROS generation by BMDMs from DNase X^−/−^ mice. Previous studies showed that *E. chaffeensis* does not block ROS generation in neutrophils, a cell type that is a potent ROS generator but is not infected by *E. chaffeensis*. Human and mouse peripheral blood neutrophils did not express DNase X. Our findings point to a unique survival mechanism of ROS-sensitive obligate intramonocytic bacteria that involves invasin EtpE binding to DNase X on the host cell surface. This is the first report of bacterial invasin having such a subversive activity on ROS generation.

## IMPORTANCE

*Ehrlichia chaffeensis* preferentially infects monocytes-macrophages and causes a life-threatening emerging tick-transmitted infectious disease called human monocytic ehrlichiosis. Ehrlichial infection, and hence the disease, depends on the ability of this bacterium to avoid or overcome powerful microbicidal mechanisms of host monocytes-macrophages, one of which is the generation of ROS. Our findings reveal that an ehrlichial surface invasin, EtpE, not only triggers bacterial entry but also blocks ROS generation by host macrophages through its host cell receptor, DNase X. As ROS sensitivity is an Achilles’ heel of this group of pathogens, understanding the mechanism by which *E. chaffeensis* rapidly blocks ROS generation suggests a new approach for developing effective anti-infective measures. The discovery of a ROS-blocking pathway is also important, as modulation of ROS generation is important in a variety of ailments and biological processes.

## INTRODUCTION

*Ehrlichia chaffeensis* is an obligatory intracellular bacterial pathogen that causes human monocytic ehrlichiosis, an emerging tick-borne zoonosis (1). The primary target cells of *E. chaffeensis* are monocytes-macrophages, which are professional phagocytes equipped with an array of oxygen-dependent and -independent microbicidal activities (2). *E. chaffeensis* replicates in a membrane-bound compartment resembling an early endosome, as it contains early endosome antigen 1, Rab5, and transferrin receptor but not lysosomal markers (3). Generation of reactive oxygen species (ROS) by the phagocyte NADPH oxidase (NOX2) complex upon pathogen encounter is a classic oxygen-dependent antimicrobial defense mechanism of phagocytes (4). Superoxide anion (O_2_^−^) serves as the starting material for the production of powerful microbicidal ROS, including hydrogen peroxide (H_2_O_2_), oxidized halogens, hydroxyl radicals, and singlet oxygen. Enzymatic detoxification of ROS to less toxic species by microbial catalases, superoxide dismutases, and peroxidases is a well-known strategy used by bacterial pathogens such as *Helicobacter*, *Salmonella*, and *Haemophilus* (5–8). Peptide methionine sulfoxide reductase of *Mycobacterium* can offer protection from oxidative damage (9). The *Mycobacterium tuberculosis* proteasome plays a major role in bacterial protection of oxidative and nitrosative damage by degrading damaged proteins (10). The carotenoid pigment of *Staphylococcus aureus* exhibits quenching activity toward singlet oxygen and offers a survival advantage relative to a mutant without this pigment against oxidative killing (11). Of the many mechanisms evolved by microbes to circumvent ROS-induced damage and killing, *E. chaffeensis* does not seem to possess the ability of enzymatic detoxification, free-radical scavenging, postexposure damage repair, oxidative stress response, or iron sequestration, as its genome does not contain such genes (12, 13). Consequently, isolated *E. chaffeensis* is quite sensitive to ROS and loses its infectivity upon exposure (13).

The phagocyte NADPH oxidase, a multicomponent enzyme, is composed of a membrane-bound heterodimeric cytochrome *b*_558_ component (gp91^*phox*^ [NOX2] and p22^*phox*^), three cytosolic subunits (p67^*phox*^, p47^*phox*^, and p40^*phox*^), and the low-molecular-weight GTPase RAC1/2 (14). When phagocytes are in a resting state, the NADPH oxidase remains inactive by keeping its components dissociated. Stimulating agents such as phorbol myristate acetate (PMA), invading pathogens, or latex beads (15) can induce the rapid assembly of all components of the NOX2 complex into a holoenzyme to catalyze the production of O_2_^−^ from oxygen. NOX2 complex activation occurs as early as 1 min in neutrophils and 5 min in monocytes (16, 17) after interaction with soluble or particulate components such as PMA, IgG-coated surfaces, oil droplets, latex particles, phospholipase C, complement fragments C5a, opsonized zymosan, bacterial lipopolysaccharide, and N-formyl peptides (18). The NOX2 complex is activated and assembled on phagosomal membranes during phagocytosis (15). *E. chaffeensis* lacks lipopolysaccharide and peptidoglycan, which typically activate the NOX2 complex of human monocytes (13). Furthermore, *E. chaffeensis* actively blocks O_2_^−^ generation by human monocytes in response to PMA (13). In addition, an *E. chaffeensis* heat-labile component induces the degradation of p22^*phox*^ to prevent the activation of NADPH oxidase, and *E. chaffeensis* inclusions do not assemble the NOX2 complex on its membrane (13). Thus, suppression of ROS production appears to be the main strategy adopted by *E. chaffeensis* to avoid this mechanism of innate defense by monocytes (19). Brief trypsin pretreatment of human monocytes prevents the inhibition of ROS generation in response to PMA (13), suggesting that surface-exposed host proteins are required for active inhibition of ROS generation. However, the bacterial and host molecules responsible for this inhibition have not been elucidated.

*E. chaffeensis* uses its surface protein entry-triggering protein of *Ehrlichia* (EtpE) to trigger entry into host cells by using the mammalian cell surface glycosylphosphatidylinositol (GPI)-anchored protein DNase X as its receptor (20). Given that *E. chaffeensis* is easily killed upon exposure to ROS (13), it seems crucial for the bacteria to enter cells in a stealthy manner without activating the cell and thus to avoid induction of ROS generation. Indeed, *E. chaffeensis* blocks NOX2 complex activation within 30 min of coincubation with human monocytes (13). Thus, we investigated whether the EtpE-triggered DNase X-mediated entry route is able to block NOX2 complex activation. Results from the present study revealed that, indeed, this route of entry actively impeded NOX2 complex activation on host cells in response to PMA to ensure successful *E. chaffeensis* colonization of macrophages.

## RESULTS

### *E. chaffeensis* inhibition of ROS generation in response to PMA is DNase X dependent.

**FIG 1:**
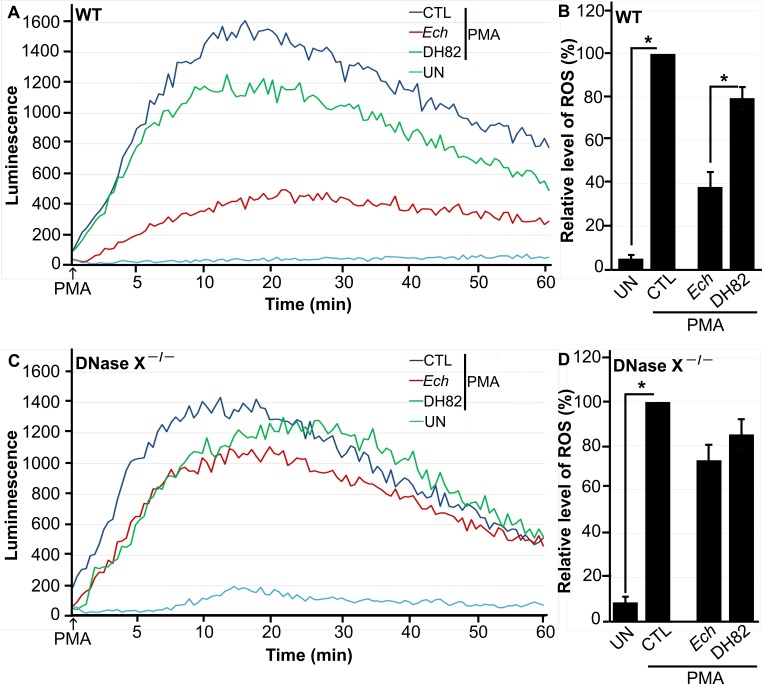
*E. chaffeensis* blocks PMA-induced ROS generation by BMDMs from WT mice but not by BMDMs from DNase X^−/−^ mice. BMDMs from WT (A, B) and DNase X^−/−^ (C, D) mice were preincubated with luminol in HBSSd for 15 min and then incubated with *E. chaffeensis* (*Ech*) isolated from infected DH82 cells, DH82 cell lysate, or HBSSd (control [CTL]) at 37°C for 30 min. ROS generation was continuously recorded as the relative chemiluminescence of oxidized luminol after the addition of PMA (0.5 µg/ml, indicated by arrows) (A, C). UN, unstimulated BMDMs in HBSSd without PMA addition. The area under the curve was measured over 60 min after PMA addition and is shown relative to ROS generation in the control with PMA, which was considered 100% (B, D). Results are presented as the mean ± the standard deviation from at least three independent experiments and were compared with a Student *t* test; *, *P* < 0.05.

Because *E. chaffeensis* enters host cells via DNase X to successfully establish infection (20), we first investigated whether the DNase X-mediated entry pathway prevents ROS generation by using a luminol-dependent chemiluminescence (LDCL) assay. The LDCL assay measures total (intra- and extracellular) O_2_^−^ and H_2_O_2_ production by using luminol, a small, membrane-permeating, luminogenic molecule (21). Mouse BMDMs were used here because (i) these cells generate ROS to an extent similar to that of human macrophages (22), (ii) *E. chaffeensis* readily infects BMDMs in an EtpE- and DNase X-dependent manner (20), and (iii) DNase X^−/−^ mice are available (20) to investigate the role of DNase X in the blockade of ROS generation. We measured levels of ROS generated in response to PMA by wild-type (WT) and DNase X^−/−^ BMDMs preincubated with isolated *E. chaffeensis* for 30 min. Canine macrophage DH82 cell lysate was added as a negative control, as *E. chaffeensis* is cultivated in DH82 cells and the complete removal of host cell components from purified bacteria is not possible. DH82 cells were used because this cell line has been used for all published successful culturing of *E. chaffeensis* strains from human monocytic ehrlichiosis patients (23–29). Similar to human peripheral blood-derived macrophages (13), mouse BMDMs generated profound ROS upon PMA treatment (Fig. 1A and B). Preincubation of BMDMs from WT mice with *E. chaffeensis* for 30 min significantly reduced PMA-induced ROS generation. However, preincubation of BMDMs from DNase X^−/−^ mice with *E. chaffeensis* for 30 min did not block PMA-induced ROS generation (Fig. 1C and D), indicating that DNase X is critical for the inhibition of PMA-induced NOX2 complex activation by *E. chaffeensis*. There is a slight diminution of ROS by DH82 lysates plus PMA compared to PMA alone, which is likely due to the nonenzymatic and enzymatic antioxidants present in the DH82 cell lysates.

### EtpE-coated latex beads block ROS generation by WT, but not DNase X^−/−^, BMDMs.

**FIG 2:**
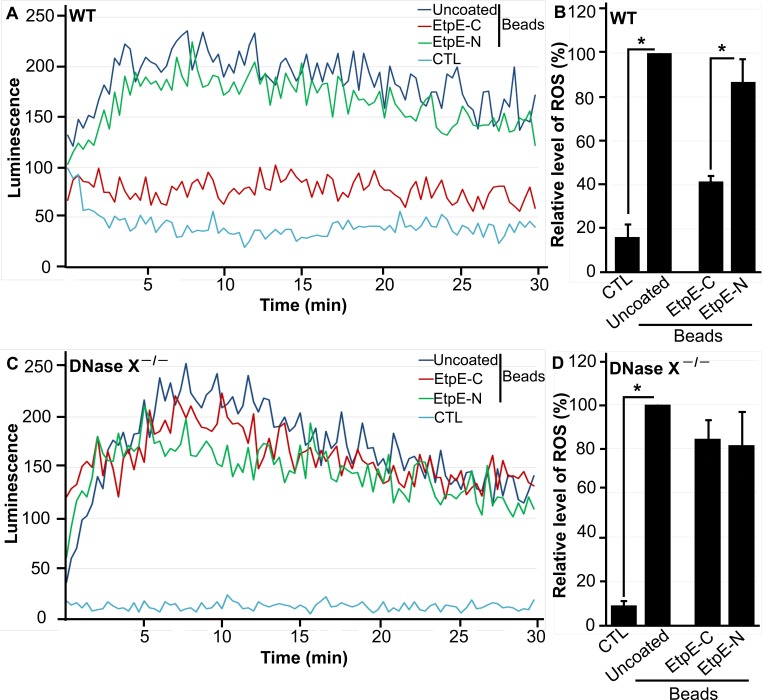
EtpE-C prevents latex bead-induced ROS generation by BMDMs from WT, but not DNase X^−/−^, mice. BMDMs from WT (A, B) and DNase X^−/−^ (C, D) mice were preincubated with luminol in HBSSd for 15 min and then incubated with 40 ng of EtpE-C- or EtpE-N-coated or uncoated beads (~5 × 10^6^) or HBSSd (control [CTL]), and ROS generation was recorded as the relative chemiluminescence of oxidized luminol (A, C). The area under the curve was measured over 30 min after bead addition and is shown relative to ROS generation with uncoated beads, which was considered 100% (B, D). Results are presented as the mean ± the standard deviation of at least three independent experiments and were compared with a Student *t* test; *, *P* < 0.05.

The C terminus of EtpE (EtpE-C) directly binds DNase X to trigger *E. chaffeensis* infectious entry (20). Although inert latex beads (similar in size to *E. chaffeensis* bacteria) that are either uncoated or coated with recombinant N-terminal EtpE protein (rEtpE-N) are phagocytosed by BMDMs in a DNase X-independent manner, beads that are coated with rEtpE-C enter into phagocytes in a DNase X-dependent manner (20). We therefore examined the effects of beads coated with EtpE-C on DNase X-dependent inhibition of ROS generation and used rEtpE-N-coated and uncoated beads as negative controls. Uncoated beads induced weaker ROS generation in both WT and DNase X^−/−^ BMDMs relative to PMA stimulation (Fig. 2A to D). Although rEtpE-C or rEtpE-N coating induced similar levels of ROS generation by DNase X^−/−^ BMDMs, rEtpE-C coating significantly reduced ROS generation by WT BMDMs relative to rEtpE-N coating (Fig. 2A to D).

**FIG 3:**
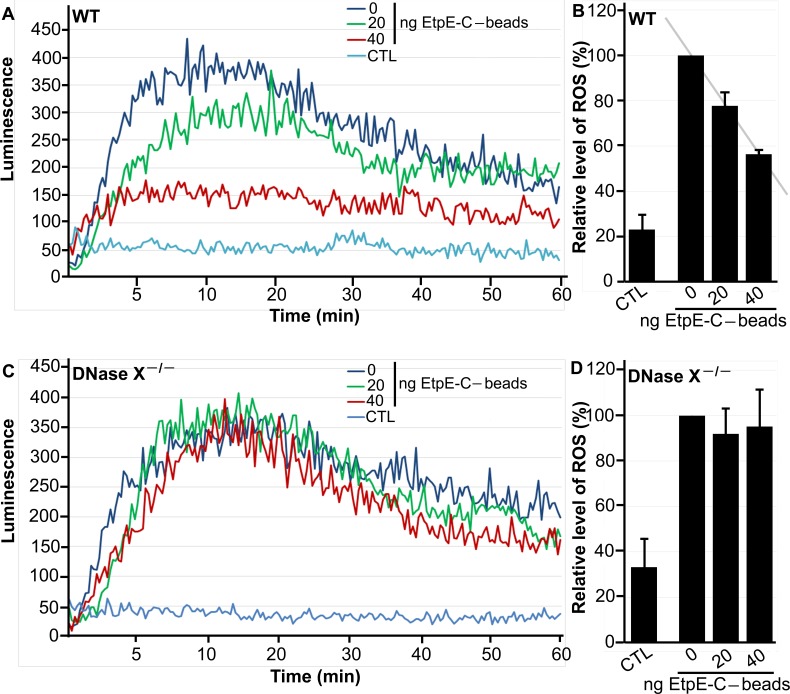
EtpE-C prevents latex bead-induced ROS generation by BMDMs from WT mice in a dose-dependent manner. BMDMs from WT (A, B) and DNase X^−/−^ (C, D) mice were preincubated with luminol in HBSSd for 15 min and then incubated with beads coated with 40, 20, or 0 ng of EtpE-C (~5 × 10^6^) or with HBSSd (control [CTL]), and ROS generation was recorded as the relative chemiluminescence of oxidized luminol (A, C). The area under the curve was measured over 60 min after bead addition and is shown relative to ROS generation with uncoated (0 ng of EtpE-C) beads, which was considered 100% (B, D). Results are presented as the mean ± the standard deviation of at least three independent experiments. The coefficient of correlation (*r* value) between the relative levels of ROS and the amounts of EtpE-C is 0.999 (*P* < 0.05).

This reduction of ROS generation by WT BMDMs was dose dependent with respect to rEtpE-C (Fig. 3A and B), whereas in DNase X^−/−^ BMDMs, the amount of rEtpE-C coating did not affect ROS generation (Fig. 3C and D). These results indicate that latex beads by themselves weakly activate the NOX2 complex in a DNase X-independent manner and that the rEtpE-C coating of beads prevents activation in a rEtpE-C dose-dependent and DNase X-dependent manner.

### EtpE-C-coated beads block PMA-induced ROS generation in WT, but not DNase X^−/−^, BMDMs.

**FIG 4:**
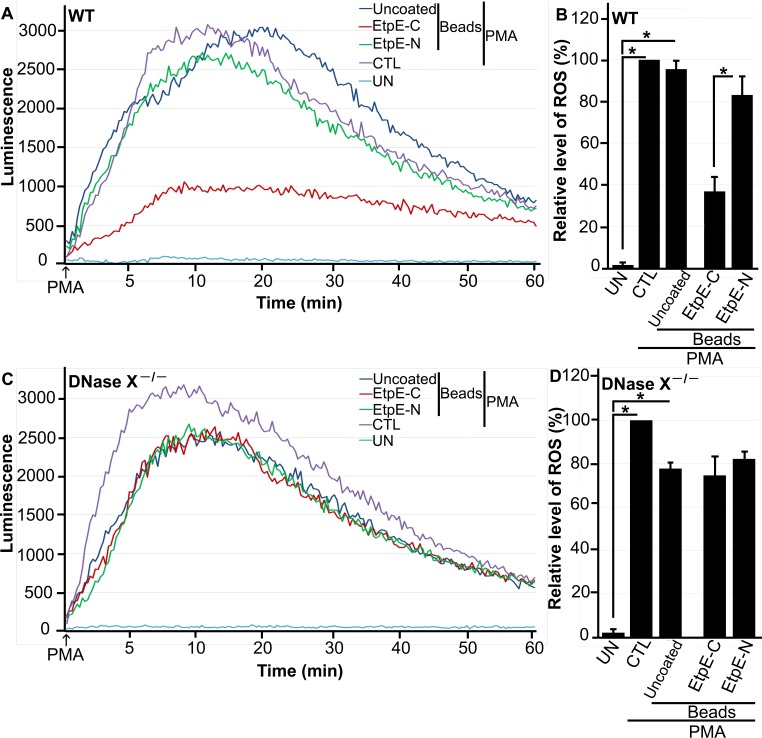
EtpE-C-coated beads block PMA-induced ROS generation by BMDMs from WT mice but not by BMDMs from DNase X^−/−^ mice. BMDMs from WT (A, B) and DNase X^−/−^ (C, D) mice were preincubated with luminol in HBSSd for 15 min and then incubated with beads coated with 40 ng of EtpE-C or EtpE-N, uncoated beads (~5 × 10^6^), or with HBSSd (control [CTL]) at 37°C for 30 min. ROS generation was induced with PMA, recorded (A, C), and analyzed (B, D), and the results are presented as in Fig. 1; UN, unstimulated BMDMs in HBSSd without PMA addition. *, *P* < 0.05.

Given that the rEtpE-C coating blocks bead-induced ROS generation by BMDMs in a DNase X-dependent manner, we examined whether preincubation of rEtpE-C-coated beads with BMDMs can block subsequent PMA-induced ROS generation analogous to *E. chaffeensis* bacteria. Regardless of whether the cells were preincubated with beads or not, PMA treatment induced significant ROS generation in both WT and DNase X^−/−^ BMDMs (Fig. 4A to D). Compared with control rEtpE-N-coated beads, rEtpE-C-coated beads significantly reduced PMA-induced ROS generation by WT BMDMs but not DNase X^−/−^ BMDMs (Fig. 4A to D). These results indicate that EtpE-C engagement with the DNase X receptor is critical for blocking PMA-induced ROS generation.

### rEtpE-C-coated bead entry is not required for inhibition of PMA-induced ROS generation by BMDMs.

**FIG 5:**
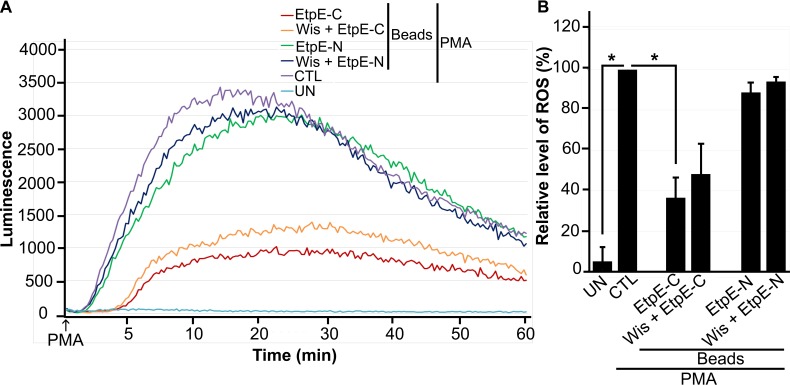
Entry of rEtpE-C-coated beads into WT BMDMs is not required for inhibition of ROS generation in response to PMA. BMDMs from WT mice were preincubated with luminol with or without 10 µM wiskostatin for 30 min at 37°C and then incubated with beads coated with 40 ng of EtpE-C or EtpE-N (~5 × 10^6^ beads) or with HBSSd (control [CTL]) at 37°C for 30 min. ROS generation was induced with PMA, recorded (A), and analyzed (B), and the results are presented as in Fig. 1; UN, unstimulated BMDMs in HBSSd without PMA addition. *, *P* < 0.05.

Maximum inhibition of PMA-induced ROS generation by *E. chaffeensis* requires ~30 min of preincubation of *E. chaffeensis* with human peripheral blood-derived macrophages (13). Thus, we examined whether EtpE-C-dependent entry is required for inhibition of PMA-induced ROS generation. DNase X receptor-dependent entry of *E. chaffeensis* and of rEtpE-C-coated beads into mammalian host cells requires actin polymerization and activation of an actin nucleation-promoting factor, neuronal Wiskott-Aldrich syndrome protein (N-WASP) (30). The cell-permeating chemical inhibitor wiskostatin binds to the GTPase-binding domain of N-WASP and thereby stabilizes its autoinhibited closed conformation (31). Pretreatment with 10 µM wiskostatin results in nearly complete inhibition of *E. chaffeensis* infection and *in vitro* DNase X-dependent actin polymerization induced by rEtpE-C (30). After pretreatment of WT BMDMs with 10 µM wiskostatin for 30 min, rEtpE-C- or rEtpE-N-coated beads were added, the mixture was incubated for 30 min more, and temporal ROS generation in response to PMA was determined. Wiskostatin treatment did not have significant effects on the inhibition of PMA-induced ROS generation by rEtpE-C-coated beads (Fig. 5A and B). Hence, EtpE-C and DNase X ligation is sufficient and EtpE-C-triggered entry is not required for inhibition of PMA-induced ROS generation.

### Human and mouse peripheral blood neutrophils do not express DNase X.

**FIG 6:**
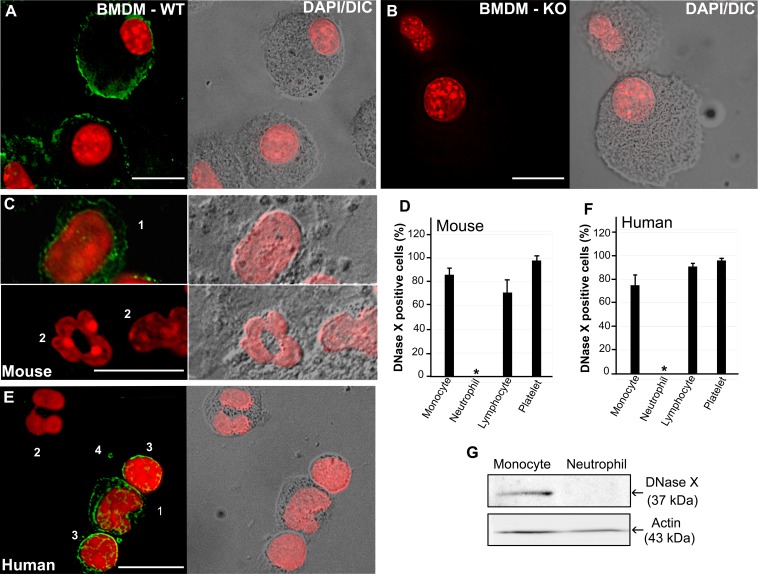
Human and mouse peripheral blood neutrophils do not express DNase X. Immunofluorescence labeling (A to C, E) and quantification (D, F) of WT and DNase X^−/−^ BMDMs and human and WT mouse PBLs with antibodies against DNase X is shown. WT BMDMs (A), DNase X^−/−^ (KO) BMDMs (B), WT mouse PBLs (C and D), and human peripheral blood leucocytes (E and F) were used. Cells: 1, monocyte; 2, neutrophil; 3, lymphocyte; 4, platelet. DeltaVision deconvolution fluorescence microscopy/differential interference contrast microscopy was used. Scale bars, 10 µm. Nuclei were stained with DAPI (pseudocolored red). (D and F) DNase X-expressing cells were scored to analyze a total of 100 of each type of cell from three individuals. Results are presented as the mean ± the standard deviation of three individuals and were compared by analysis of variance; *, *P* < 0.05. (G) Western blot analysis of human peripheral blood-derived monocytes and neutrophils with anti-DNase X and anti-actin antibodies.

*E. chaffeensis* neither infects neutrophils nor blocks their PMA-induced ROS generation (13). Although it was reported that DNase X is expressed in various human tissues and cells (32) and DNase X is clearly detected on monocytes and macrophages from humans, dogs, and mice (20), expression of DNase X by neutrophils has never been reported. Therefore, we examined DNase X expression in peripheral blood leukocytes (PBLs) by using the DNase X-specific antibody. As a control, DNase X protein was undetectable in DNase X^−/−^ BMDMs, whereas DNase X was clearly detectable on WT BMDMs by immunofluorescence labeling (Fig. 6A). Immunofluorescence labeling showed that DNase X protein was undetectable in both mouse and human peripheral blood neutrophils, whereas DNase X was clearly detectable on monocytes, lymphocytes, and platelets from the same blood specimens (Fig. 6B to E). Western blot analysis corroborates the lack of DNase X protein expression by human peripheral blood neutrophils (Fig. 6G). Taken together, the results show that mature neutrophils lack surface DNase X, which is required for EtpE-mediated entry and blockade of ROS generation by *E. chaffeensis*.

### *E. chaffeensis* induces cytochrome *b* light chain (p22^*phox*^) degradation in BMDMs in a DNase X-dependent manner, but EtpE-C-coated beads do not induce p22^*phox*^ degradation.

**FIG 7:**
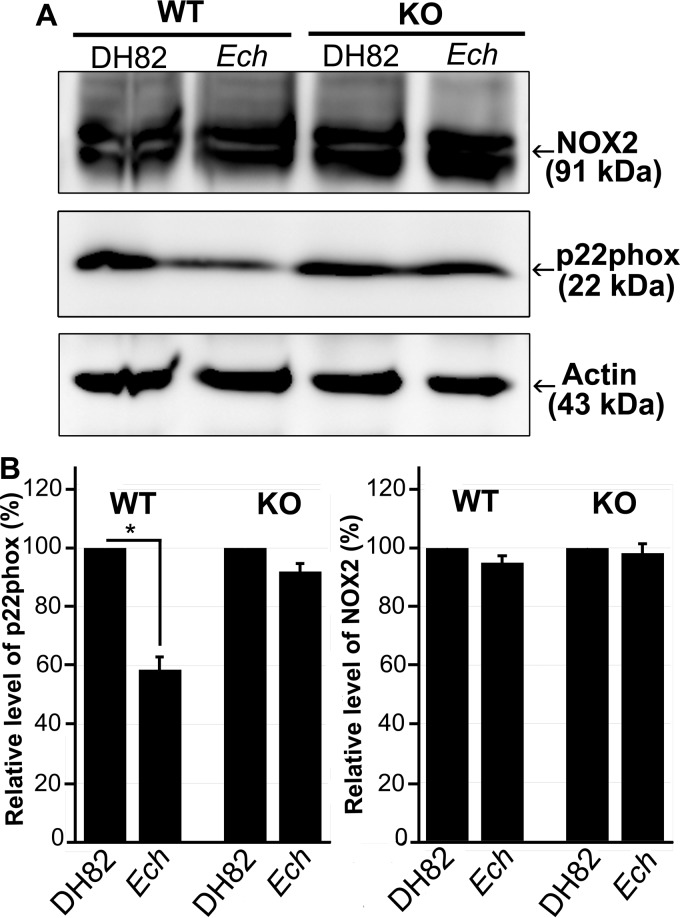
Reduction of p22^*phox*^ by *E. chaffeensis* in BMDMs from WT mice but not by BMDMs from DNase X^−/−^ (KO) mice. (A) BMDMs from WT and DNase X^−/−^ mice were incubated with DH82 cell lysate or host cell-free *E. chaffeensis* (*Ech*) at 37°C for 2 h. Whole-cell lysates were prepared and subjected to Western blotting with antibodies against NOX2, p22^*phox*^, and actin. (B) The relative amounts of p22^*phox*^ and NOX2 were calculated by normalizing the band intensities with actin, and the ratio observed in the control group (DH82 cell lysate) was arbitrarily set to 100%. Results are presented as the mean ± the standard deviation of at least three independent experiments and were compared with a Student *t* test; *, *P* < 0.05.

**FIG 8:**
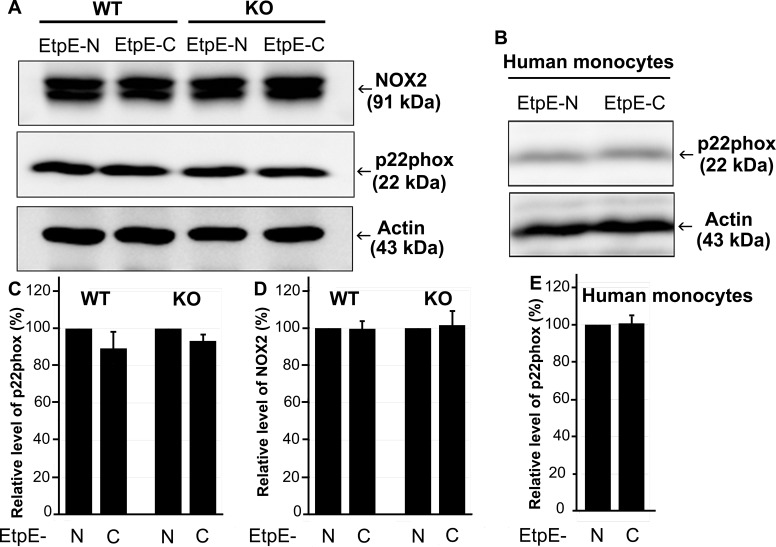
EtpE-C-coated beads do not induce reduction of p22^*phox*^ in BMDMs from WT or DNase X^−/−^ (KO) mice, and human monocytes. BMDMs from WT and DNase X^−/−^ mice (A, C, D) or human peripheral blood monocytes (B, E) were incubated with EtpE-C- or EtpE-N-coated beads at 37°C for 2 h. Whole-cell lysates were prepared and subjected to Western blotting with antibodies against NOX2, p22^*phox*^, and actin. (C to E) Relative amounts of p22^*phox*^ and NOX2 were calculated by normalizing the band intensities with actin, and the ratio observed in the control group (incubated with EtpE-N-coated beads) was arbitrarily set to 100. Results are presented as the mean ± the standard deviation of at least three independent experiments.

*E. chaffeensis* induces rapid degradation of p22^*phox*^ in human peripheral blood-derived macrophages (13). Therefore, we examined whether EtpE-C and DNase X have a role in the degradation of p22^*phox*^. Our results showed that *E. chaffeensis* induces p22^*phox*^ degradation in a DNase X-dependent manner in mouse BMDMs (Fig. 7). However, rEtpE-C-coated beads did not induce significant p22^*phox*^ degradation in mouse BMDMs or human peripheral blood monocytes (Fig. 8), although rEtpE-C coating did block PMA-induced ROS generation by mouse BMDMs (Fig. 4 and 5). Thus, EtpE-C--DNase X ligation alone is not sufficient for p22^*phox*^ degradation and prior degradation of p22^*phox*^ is not required for rEtpE-C-mediated inhibition of PMA-induced ROS generation by BMDMs or human peripheral blood monocytes.

## DISCUSSION

The present study indicates that EtpE is a crucial bacterial molecule not only for *E. chaffeensis* host cell entry (20) but also to avoid induction of ROS generation. Several microbial products are known to block NOX2 complex activation. Secreted *M. tuberculosis* nucleoside diphosphate kinase binds and inactivates RAC1, leading to defective NOX2 complex assembly and ROS generation (33). Listeriolysin O suppresses phospholipase C-mediated activation of the NOX2 complex by inhibiting the proper localization of its components in the phagosome (34). The tyrosine phosphatase YopH, which is a type III secretion system effector of *Yersinia pseudotuberculosis*, inhibits Fc receptor-mediated ROS generation in macrophages, and its phosphatase activity is required for this suppression (35). Hemozoin pigment of *Plasmodium* inhibits ROS generation by monocytes, although the mechanism is unknown (36). EtpE is an ehrlichial surface-anchored outer membrane protein, and its ROS-inhibitory mechanism is distinct from the aforementioned previously characterized mechanisms. Notably, this EtpE-C-mediated blockade of NOX2 complex activation represents a global inhibition, as even PMA-induced NOX2 complex activation is blocked in its presence (13).

Several intracellular pathogens enter phagocytes via receptors that do not activate host cells. Complement receptor type 3 is such a receptor, and it is used by multiple pathogens, including *Mycobacterium* (37, 38), *Listeria monocytogenes* (39), *Rhodococcus equi* (40), *Legionella pneumophila* (41), and *Leishmania donovani* (42). The present study revealed that entry mediated by the host surface protein DNase X not only does not activate formation of the NOX2 complex but also rather actively blocks its activation, as does *E. chaffeensis*. DNase X-dependent inhibition corroborates our previous finding that a host cell surface protein is required for the ROS blockade by *E. chaffeensis* (13). Thus, EtpE-C ligation of DNase X transduces at least two distinct signals in host cells, one to spatiotemporally polymerize actin for bacterial internalization (30) and another to block NOX2 complex activation.

The *E. chaffeensis* and EtpE-C-coated bead entry pathway involves EtpE-C ligation of DNase X, which recruits and binds human transmembrane glycoprotein CD147 (basigin), cytoplasmic and nuclear heterogeneous nuclear ribonucleoprotein K (hnRNP-K), and N-WASP (30); all four of these human proteins are required for ehrlichial entry into host cells and establishment of infection (30). EtpE-C induces actin polymerization *in vitro* in a DNase X- and N-WASP-dependent manner (30). The present study revealed that N-WASP activation is not required for the blockade of NOX2 complex activation. Whether CD147 and/or hnRNP-K are involved in the inhibition of NOX2 complex activation remains to be investigated. Whether any other molecule that binds DNase X, such as DNA, can also block PMA-induced ROS generation is currently unknown.

*E. chaffeensis* induces rapid destabilization/degradation of p22^*phox*^ in human monocytes (13). Although this phenomenon was reproducible in mouse BMDMs incubated with *E. chaffeensis* in the present study, rEtpE-C-coated beads did not induce degradation of p22^*phox*^ in BMDMs or human peripheral blood-derived macrophages. Thus, degradation likely requires other ehrlichial components and p22^*phox*^ degradation is not essential for the blockade of NOX2 complex activation.

In the present study, we were able to answer two long-standing questions concerning the absence of *E. chaffeensis* infection of peripheral blood neutrophils and the inability of *E. chaffeensis* to block ROS generation by human neutrophils (13). Both are related to the absence of DNase X expression in terminally differentiated neutrophils. *Anaplasma phagocytophilum*, a pathogen closely related to *E. chaffeensis* in the family *Anaplasmataceae*, similarly lacks enzymatic detoxification, free-radical scavenging, post-ROS exposure damage repair, an oxidative stress response, and iron sequestration (12, 13), and isolated *A. phagocytophilum* is quite sensitive to ROS (13). *A. phagocytophilum* preferentially infects granulocytes but not monocytes, and *A. phagocytophilum* has evolved mechanisms analogous to those of *E. chaffeensis* to block ROS generation during its binding of and entry into human neutrophils. Specifically, *A. phagocytophilum* blocks ROS generation in response to *Escherichia coli*, PMA, *N*-formylmethionine-leucyl-phenylalanine, or Fc-Oxyburst immune complexes in human and murine neutrophils but not in monocytes (43, 44). Despite the phylogenetic relatedness of these two bacteria, the absence of DNase X from neutrophils suggests that they use distinct signaling pathways to carry out a host cell-specific blockade of ROS generation.

Taken together, these results indicate a clear survival advantage for *E. chaffeensis* as a result of its ability to use an EtpE-C-triggered DNase X-mediated entry route over a DNase X-independent pathway, as the former provides stealthy entry without eliciting the formation of the microbicidal NOX2 complex of its host cells.

## MATERIALS AND METHODS

### Ethics statement.

All animal experiments were performed in accordance with the Ohio State University Institutional Animal Care and Use Committee guidelines and approved e-protocol. The university program has full continued accreditation by the Association for Assessment and Accreditation of Laboratory Animal Care International under 000028, dated 9 June 2000, and has Public Health Services assurance renewal A3261-01, dated 6 February 2015 through 28 February 2019. The program is licensed by the USDA, number 1-R-014, and is in full compliance with Animal Welfare Regulations. DNase X^−/−^ (20) and congenic WT C57BL/6 mice (The Jackson Laboratory, Bar Harbor, ME) were bred in the animal facilities of The Ohio State University.

### Isolation of BMDMs.

DNase X^−/−^ and WT mice (7 to 12 weeks old) were euthanized, and their femurs were removed without damaging the bone. The muscle was completely removed, and both ends of the femur were cut. The bone marrow was flushed out with 10 ml of RPMI 1640 medium (Mediatech, Manassas, VA), and the resulting cells were centrifuged in a Sorvall T6000D centrifuge at 450 × *g* for 5 min. Red blood cells were lysed with 5 ml of ACK (ammonium-chloride-potassium) lysis buffer (0.15 M NH_4_Cl, 10 mM KHCO_3_, 0.1 mM Na_2_EDTA) for 3 min, after which the tube was filled up with 15 ml of RPMI 1640 medium and centrifuged at 450 × *g* for 5 min. The supernatant was discarded, and cells were cultured in T75 flasks (5 × 10^7^ cells/flask) containing RPMI 1640 medium with 10% fetal bovine serum (FBS; Atlanta Biologicals, Lawrenceville, GA), 1% l-glutamine (Gibco, Grand Island, NY), 10% conditioned medium from L929 cells (ATCC, Manassas, VA), and 1% antibiotic-antimycotic mixture (100×; Gibco) at 37°C in 5% CO_2_ and 95% air in a humidified atmosphere as previously described (45). After 3 days, fresh medium (RPMI with 10% FBS, 1% l-glutamine, 10% L929 cell-conditioned medium, and 1% antibiotic-antimycotic mixture) was added to the attached cells, and on day 6, BMDMs were plated at 5 × 10^5^/well in sterile 96-well solid white polystyrene microplates (Thermo Fisher, Waltham, MA) for overnight incubation. BMDMs were verified by a phagocytosis assay (46) by plating 3 × 10^5^ cells in a 24-well plate and culturing them overnight at 37°C. Carboxylate-modified latex beads (2.0 µm; Sigma-Aldrich, St. Louis, MO) were added at 6 × 10^7^/well, and the plates were incubated at 37°C for 30 min. The treated cells were washed five times with phosphate-buffered saline (PBS; 137 mM NaCl, 2.7 mM KCl, 10 mM Na_2_HPO_4_, 2 mM KH_2_PO_4_), trypsinized with TrypLE Express (GIBCO), and stained with Hema 3 stain (Thermo Fisher) after centrifugation in a cytospin 4 cytocentrifuge (Thermo Fisher). One hundred cells were scored, and the number of phagocytic cells was determined to calculate the percentage of macrophages. Mouse BMDMs after incubation in 10% L929 cell-conditioned medium for 7 days consisted of 87% ± 2% macrophages.

### Isolation of recombinant proteins.

rEtpE-C and rEtpE-N were produced and purified as previously described (20). Briefly, *E. coli* BL21 cells transformed with an EtpE-C or EtpE-N plasmid were induced with 0.5 mM isopropyl-β-d-thiogalactopyranoside at 30 or 37°C, respectively, for 5 h, after which they were centrifuged, harvested, and lysed. After several washings, the insoluble inclusion of rEtpE-C or rEtpE-N protein was dissolved in 6 mM guanidine hydrochloride or 8 M urea, respectively, and loaded onto a cobalt affinity column consisting of HisPur Cobalt Resin (Thermo Fisher). The column was washed with 10 mM imidazole in 6 M urea, and bound proteins were eluted with 250 mM imidazole in 6 M urea.

### Coating of latex beads with proteins.

Carboxylate-modified latex beads (0.5 µm) were coated with rEtpE-C and rEtpE-N solubilized in 6 M urea as previously described (20) at a ratio of 40 ng of protein/5 × 10^6^ beads. Then, 50 µl of 25 mM 2-(*N*-morpholino)ethanesulfonic acid (MES) buffer, pH 6.0 (Sigma-Aldrich), was added every 5 min until a total volume of 100 times that of the urea solution was reached. Protein-coated beads were incubated for 30 min at 25°C, and the coated beads were harvested with a PerfectSpin 24R microcentrifuge (Peqlab, Stanwood, WA) at 14,000 × *g* for 2 min. The MES buffer was completely removed, and HBSSd, consisting of phenol red-free Hanks’ balanced salt solution (Sigma-Aldrich) supplemented with 2 mg/ml dextrose (Hospira, Lake Forest, IL), was added to the coated beads and the mixture was slightly sonicated with an Ultrasonic W-380 Sonicator (Heat Systems-Ultrasonics, Inc., Farmingdale, NY). Uncoated beads were processed in a similar manner. Protein-coated and uncoated beads were treated with 20 µg/ml polymyxin B sulfate (Sigma-Aldrich) to neutralize possible endotoxin contamination (47). The coating of beads was confirmed by dot blot assay with peroxidase-conjugated mouse anti-polyhistidine monoclonal antibody (Sigma-Aldrich) as previously described (20).

### Isolation of host cell-free *E. chaffeensis*.

*E. chaffeensis* Arkansas (23) was cultured in the canine macrophage cell line DH82 (48) in Dulbecco’s minimal essential medium (DMEM; Mediatech) supplemented with 5% FBS and 2 mM l-glutamine at 37°C in 5% CO_2_ and 95% air in a humidified atmosphere as previously described (49). Cells were monitored for 2 to 3 days for infection by using Hema 3 stain on centrifuged specimens and were passaged or harvested when the percentage of infected cells reached >95% as previously described (49). *E. chaffeensis-*infected cells (~1 × 10^8^ cells >90% infected from two T75 flasks) were harvested by centrifugation at 400 × *g* for 5 min. The pellet was resuspended in DMEM and sonicated on ice for 8 s at an output setting of 2 with a W-380 Sonicator (Heat Systems, Newtown, CT). Unbroken cells were removed by centrifugation at 1,000 × *g* for 5 min. The supernatant was passed through 5.0- and 2.7-µm GD/X nylon filters (Whatman, Florham Park, NJ) to remove cell debris, and centrifuged at 10,000 × *g* for 10 min as previously described (50). The resulting bacterial pellet was resuspended in HBSSd.

### Quantification of *E. chaffeensis*.

DNA was purified from host cell-free *E. chaffeensis* bacteria with a QIAamp DNA minikit (Qiagen, Germantown, MD). To quantify *E. chaffeensis*, an absolute quantification method was used by creating a standard curve of the *E. chaffeensis* 16S rRNA gene copy number by quantitative PCR (qPCR) in accordance with the manufacturer’s protocol (Stratagene, Waltham, MA) as previously described (50). The qPCR mixture (20 μl) included 500 ng of DNA, 0.25 μM each primer, and 10 μl of SYBR green qPCR master mix (Thermo Fisher). Primer sequences for the *E. chaffeensis* 16S rRNA gene are shown in reference 45. PCR was performed in the Mx3000P instrument (Stratagene). Twofold dilutions (0 to 1:16) of the standard template (*E. chaffeensis* 16S rRNA gene cloned into vector pUC19) were prepared, and a qPCRs were performed to generate a standard curve by plotting the initial template quantity against the threshold cycle (*C*_*T*_) values for the standards. The copy number of the targeted gene in DNA samples was calculated by comparing the *C*_*T*_ value with the standard curve.

### LDCL assay.

To investigate total ROS production, we adapted an LDCL assay in the presence of horseradish peroxidase (51). BMDMs derived from DNase X^−/−^ and WT mice were cultured in 96-well assay plates at 5 × 10^5^/well. Luminol (1 mM; Sigma-Aldrich) and 4 U/ml horseradish peroxidase (Sigma-Aldrich) in 150 µl of HBSSd were added to each well and incubated at 37°C for 15 min. Into each well, 50 µl of rEtpE-C- or rEtpE-N-coated or uncoated beads at a ratio of 10/cell or additional HBSSd was added. For a dose-dependent assay, beads coated with 40, 20, or 0 ng of EtpE-C (~5 × 10^6^ beads) were added. To determine the inhibition of PMA-induced ROS generation, 50 µl of host cell-free *E. chaffeensis* at ~100 *E. chaffeensis* bacteria/cell or DH82 lysate (derived from the same number of uninfected cells by the same sonication, centrifugation, and filtration methods used for infected cells), rEtpE-C- or rEtpE-N-coated or uncoated beads, or HBSSd was added, the mixture was incubated for 30 min, and PMA (0.5 μg/ml; Sigma-Aldrich) in 50 µl of HBSSd was added. HBSSd alone was added to cells as a negative control. For wiskostatin pretreatment, WT BMDMs were seeded into 96-well plates at 5 × 10^5^/well in 150 µl of HBSSd supplemented with 10 µM wiskostatin (Sigma-Aldrich) or 10 µl of dimethyl sulfoxide, 1 mM luminol, and 4 U/ml horseradish peroxidase and incubated at 37°C for 30 min. HBSSd (50 μl) or EtpE-C- or rEtpE-N-coated beads (~5 × 10^6^) were added to the wells, the plates were incubated for 30 min more, and then 0.5 µg/ml PMA in 50 µl of HBSSd was added. HBSSd alone was added to cells as a negative control. The plate was continuously read every 20 s with a Synergy HTX Multi-Mode Reader (BioTek, Winooski, VT) beginning at the point when rEtpE-C- or rEtpE-N-coated or uncoated beads, HBSSd, or PMA was added. The area under the curve was measured for 0.5 or 1 h and reported in luminescence intensity units.

### Analysis of DNase X expression in human and mouse PBLs.

PBLs were isolated via a 10-min centrifugation at 400 × *g* of human (American Red Cross, Columbus, OH) and mouse whole blood, followed by harvesting of the buffy coat. Red blood cells were lysed with 5 ml of ACK lysis buffer for 5 min. PBLs were centrifuged and fixed with 4% paraformaldehyde for 15 min. After three washes with PBS, excess paraformaldehyde was quenched with 50 mM NH_4_Cl in PBS for 15 min and blocked with 1% bovine serum albumin in PBS. Mouse anti-DNase X monoclonal antibody (Abcam, Inc., Cambridge, MA) was diluted 1:100 in PBS, 200 µl of the diluted antibody was added to the slides, and they were incubated at 4°C overnight. The following day, the slides were washed three times with PBS, and secondary antibody, an Alexa Fluor 488-conjugated goat anti-mouse antibody (Life Technologies, Inc., Carlsbad, CA) diluted 1:100 in PBS, was added to the slides and they were incubated for 60 min at room temperature. Cells were washed three times with PBS, DAPI (4′,6-diamidino-2-phenylindole; Invitrogen, Carlsbad, CA) was added, and the cells were incubated for 3 min at room temperature. The cells were then washed three times with PBS and mounted with glycerol-PBS and observed with a DeltaVision Deconvolution Microscope (GE Healthcare Bio-Sciences, Pittsburgh, PA). Human peripheral blood monocytes and neutrophils were isolated by using a Histopaque 1077 and Histopaque 1119 (Sigma-Aldrich) density gradient as previously described (13), and 3 × 10^6^ cells were solubilized in 100 µl of SDS sample buffer and boiled for 5 min. Samples (10 µl) were separated on a 10% SDS-polyacrylamide gel, transferred to a nitrocellulose membrane (Bio-Rad, Hercules, CA), and then subjected to Western blotting with mouse anti-DNase X monoclonal antibody, and rabbit anti-actin antibody (Sigma-Aldrich) was used as a loading control. Reactive bands were visualized by enhanced chemiluminescence by incubating the membrane with the respective horseradish peroxidase-conjugated secondary antibodies and the same volume of H_2_O_2_ solution (Thermo Scientific) and Luminol/Enhancer solution (Thermo Scientific).

### Western blot analysis of p22^phox^ and NOX2.

WT and DNase X^−/−^ BMDMs were plated in RPMI with 10% FBS, 1% l-glutamine, 10% L929 cell-conditioned medium, and 1% antibiotic-antimycotic mixture at 2 × 10^6^/well in 12-well plates. Human peripheral blood monocytes isolated with Histopaque 1077 were plated in RPMI with 10% FBS and 1% l-glutamine in a similar manner. The following day, the supernatant was removed and 1 ml of host cell-free *E. chaffeensis*, the DH82 cell lysate, or rEtpE-C- or rEtpE-N-coated beads was added to the BMDMs (100 *E. chaffeensis* bacteria or beads/cell) and they were incubated for 2 h. For human monocytes, rEtpE-C- or rEtpE-N-coated beads were added. The cells were washed, and 2 µl/well protease inhibitor cocktail (EMD Millipore, Billerica, MA) was added. Cells were solubilized in SDS sample buffer and boiled for 5 min. Samples were separated on a 10% SDS-polyacrylamide gel, transferred to a nitrocellulose membrane, and then subjected to Western blotting with rabbit anti-NOX2 antibody (gift from S. Tsunawaki). Rabbit anti-actin antibody was used as a loading control. To detect p22^*phox*^, a 15% SDS-polyacrylamide gel and polyvinylidene difluoride membrane (Schleicher & Schuell Bioscience, Keene, NH) and rabbit anti-p22^*phox*^ antibody (full length; gift from S. Tsunawaki) were used for Western blotting. Reactive bands were visualized by enhanced chemiluminescence, and images were captured and densitometric analysis was performed with the LAS3000 image documentation system (FUJIFILM Medical Systems USA, Inc., Stamford, CT). Band intensities were normalized against actin in the corresponding samples.

### Statistical analysis.

Experiments were independently repeated at least three times. Statistical analysis was performed with a two-tailed Student *t* test. For experiments involving more than two groups, an analysis of variance was performed. For all tests, a *P* value of <0.05 was considered significant. All statistical analyses, including correlation analysis, were performed with Microsoft Excel 2010.
